# Supplementary material for: Diversity of bacterial small RNAs drives competitive strategies for a mutual chaperone
Source: Nat Commun. 2022 May 4;13:2449. doi: 10.1038/s41467-022-30211-z (PMC9068810; doi:10.1038/s41467-022-30211-z)
Supplement: Supplementary file 1 — Supplementary Information [file 41467_2022_30211_MOESM1_ESM.pdf]

SUPPLEMENTARY INFORMATION

Diversity of bacterial small RNAs drives competitive strategies for a mutual chaperone

Jorjethe Roca[1], Andrew Santiago-Frangos[2] and Sarah A. Woodson[1]*

[1]T. C. Jenkins Department of Biophysics, Johns Hopkins University, 3400 N. Charles St., Baltimore, MD. 21218. USA; [2]CMDB Program, Johns Hopkins University, 3400 N. Charles St., Baltimore, MD. 21218. USA

SUPPLEMENTARY FIGURES

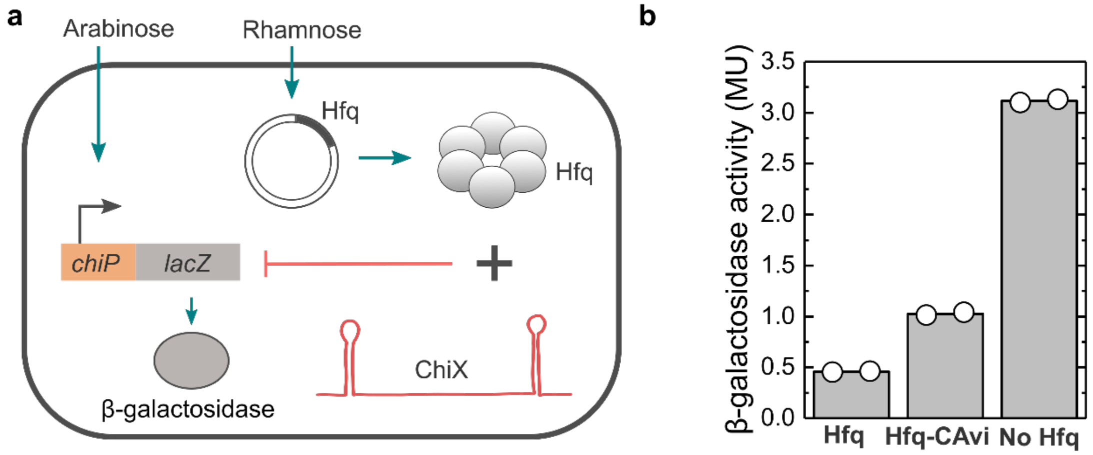

Supplementary Fig. 1. **An AviTag minimally impacts Hfq function in *E. coli.*** **a** Schematic of ChiX and *chiP-lacZ* reporter assay. To determine the impact of the C-terminal AviTag on Hfq's function in post-transcriptional gene regulation by sRNAs, we measured its effect on *chiP* down-regulation by ChiX sRNA. We chose this sRNA-mRNA regulatory pair because it is particularly sensitive to truncation of the Hfq CTD[1]. *E. coli* strain DJS2689 (PM1205 *lacI'::pBAD-chiP-lacZ Δhfq::cat-sacB*) carrying a chromosomal fusion of *chiP-lacZ* was transformed with pD871 plasmids expressing either WT Hfq or Hfq-CAvi (fully tagged), and grown in LB-Miller media with 0.004% arabinose and 0.001% rhamnose to induce expression of the *chiP-lacZ* fusion and Hfq, respectively. **b** Endogenous ChiX repression of *chiP-lacZ* expression in DJS2689 cells expressing wild type Hfq, Hfq-CAvi or no Hfq. Cells were grown to an OD_{600} of 0.7-0.8 and then assayed for β-galactosidase activity (Miller units). In the absence of Hfq, endogenously transcribed ChiX did not efficiently bind to and inhibit translation of the *chiP-lacZ* fusion, resulting in high activity. However, induction of wildtype Hfq expression enabled efficient pairing of ChiX to *chiP-lacZ* and inhibition of *lacZ* translation, resulting in nearly seven-fold lower Miller units as compared to a no-Hfq control. Induction of Hfq with a C-terminal AviTag fusion resulted in a three-fold decrease in Miller units as compared to a no-Hfq control. The symbols indicate the results of two technical replicates. Source data are provided as a Source Data file.

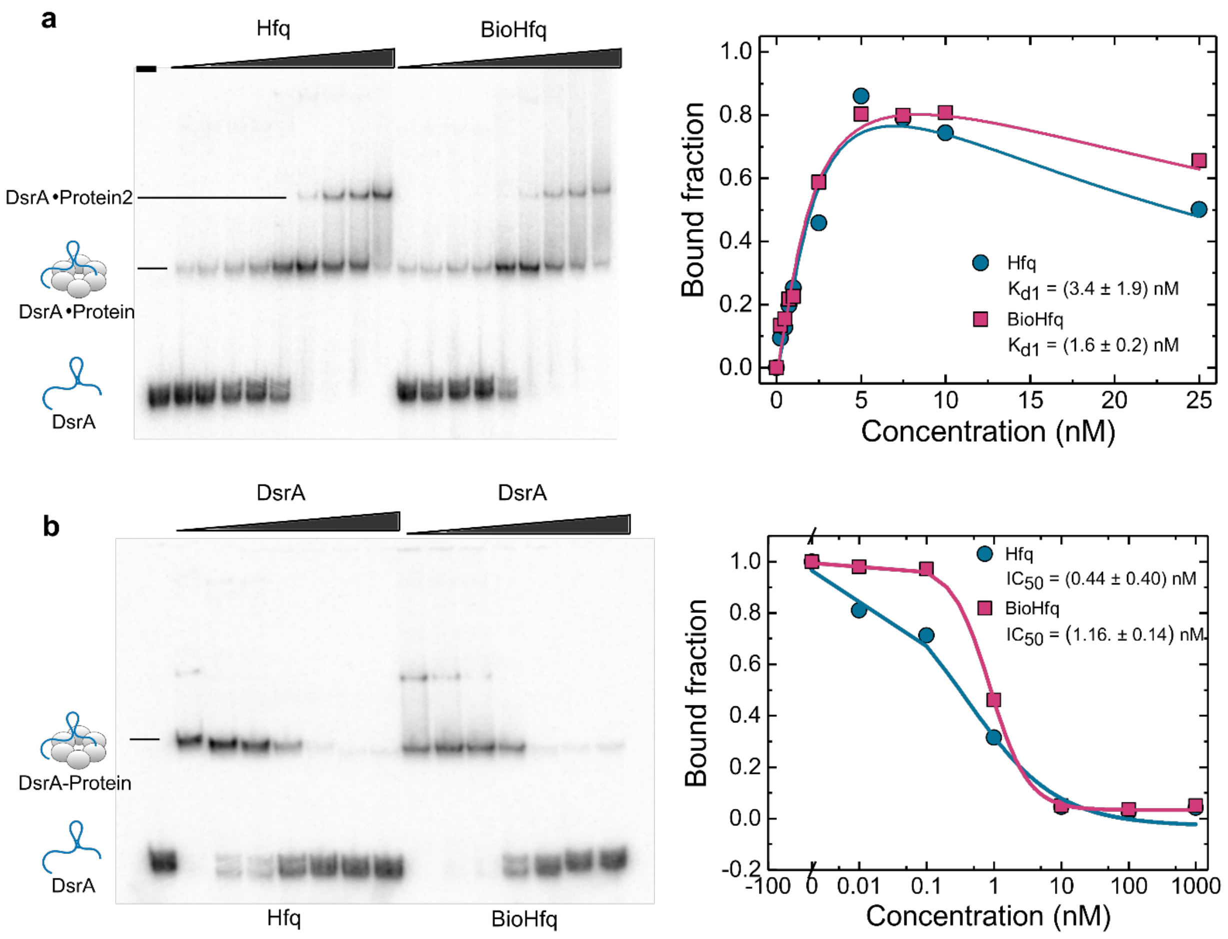

Supplementary Fig. 2. BioHfq minimally impacts sRNA binding and competition. a ^{32}P-DsrA (2 nM) binding to increasing concentrations of Hfq or BioHfq (0, 0.25, 0.5, 0.75, 1, 2.5 5, 7.5, 10 and 25 nM). Plot shows the fraction of DsrA bound to a single Hfq hexamer. A partition function for the association of one and two hexamers was fitted into the data. Data shown represent one of two independent trials. The reported K_D values are the mean and propagated errors of the fit parameters from both trials. Dissociation constants for Hfq (teal) and BioHfq (purple) differed about two-fold. **b** Displacement of ^{32}P-DsrA (2 nM) from Hfq or BioHfq (5 nM) when challenged with increasing concentrations of unlabeled DsrA (0.01, 0.1, 1, 10, 100 and 1000 nM); reported IC_{50} values are the mean of two trials as in **a**. The differences in K_D and IC_{50} reflect some impact on sRNA binding and competition by modification of the CTDs. This impact is modest compared to the differences between natural sRNAs in similar assays. Source data are provided as a Source Data file.

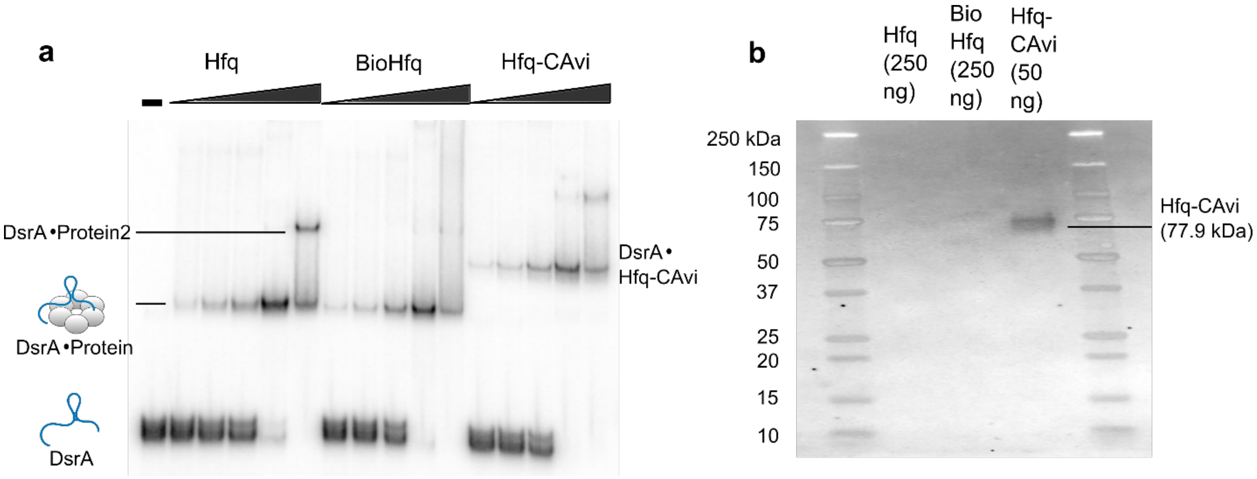

Supplementary Fig. 3. Sparse incorporation of Avi-tag into BioHfq. a ^{32}P-DsrA binding to increasing concentrations of Hfq, BioHfq (sparsely tagged) or Hfq-CAvi (fully-tagged), by EMSA. Protein concentrations were 0.25, 0.5, 1, 5 and 10 nM; lane (–), no Hfq. Complexes with Hfq-CAvi migrate more slowly in the native gel than WT Hfq complexes. BioHfq complexes migrated similarly as WT Hfq complexes, suggesting that BioHfq contains less than one Avi-tagged subunit per hexamer, on average. **b** Western blot of Hfq preparations with anti-Avi Tag antibody. The Avi-tag was undetectable in 250 ng BioHfq (center lane) compared to 50 ng Hfq-CAvi (right lane), indicating that BioHfq contains ≤10% Avi tag. For both panels, the data shown represent one of two independent trials. Assuming random incorporation of tagged subunits into Hfq hexamers during co-expression in *E. coli*, and an upper limit of 10% Avi-tagged subunits, the Poisson distribution predicts ≤ 9.05% of hexamers have a single Avi-tagged subunit. Of the hexamers that could be immobilized on the slides (*e.g.*, have at least one biotinylated subunit), no more than 4.9% are expected to have more than one tag. Source data are provided as a Source Data file.

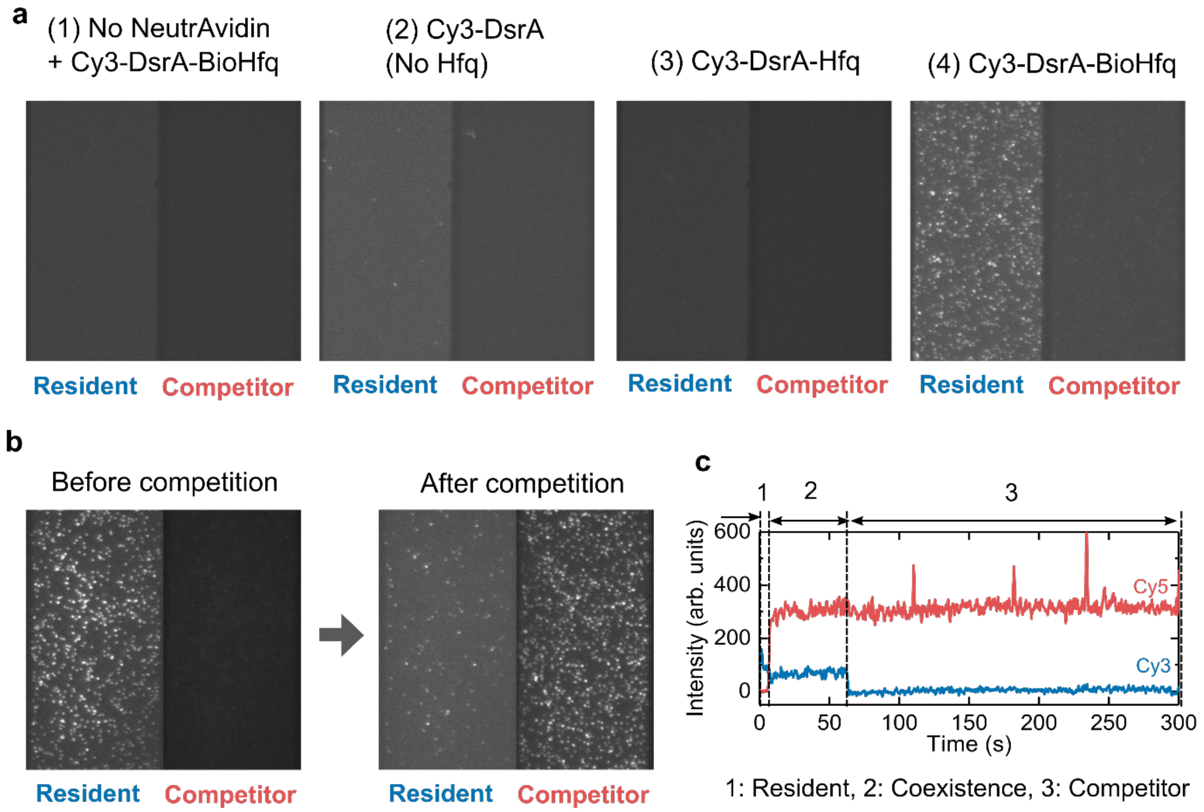

Supplementary Fig. 4. sRNA·BioHfq complexes are specifically immobilized and subject to competition by other sRNAs. a Controls for the specificity of sRNA immobilization. Representative field of view on the microscope slide with immobilized single molecules when adding (1) Cy3-DsrA·BioHfq complexes to a slide prepared without NeutrAvidin, (2) Cy3-DsrA only (no Hfq), (3) Cy3-DsrA·Hfq complexes and (4) Cy3-DsrA·BioHfq complexes. Immobilized Cy3 spots were only observed for Cy3-DsrA·BioHfq complexes on slides coated with NeutrAvidin. Left, Cy3 channel (residents); right, Cy5 channel (competitors). **b** Representative field of view with Cy3-sRNA·BioHfq complexes, before and 5 min after the addition of Cy5-labeled competitor sRNAs. Since BioHfq is immobilized, only bound sRNAs are observed. **c** Representative single-molecule trace showing binding intervals analyzed in this study: (1) Resident only before competitor binding, (2) Coexistence of resident and competitor, and (3) Competitor only. Fluorescence intensities were recorded for 3,000 frames (100 ms/frame) using an alternating excitation scheme (see Methods). Source data are provided as a Source Data file.

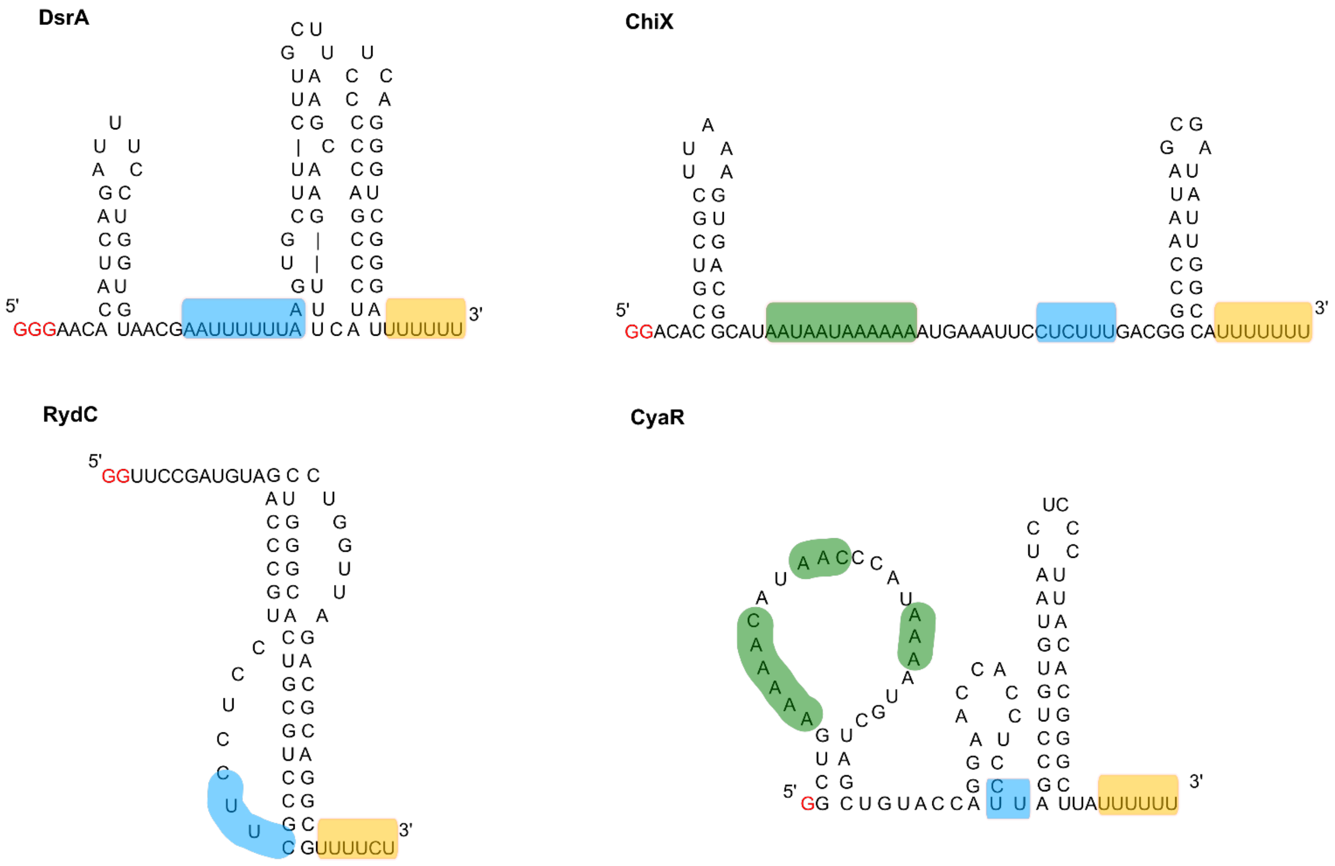

Supplementary Fig. 5. **Secondary structures of the sRNAs used in this study.** The structures of DsrA[2,3], ChiX[4] and RydC (from *Salmonella enterica*)[5,6] have been verified experimentally or through bioinformatics. The structure of CyaR was predicted using Mfold[7]. RydC from *S. enterica* was chosen over *E. coli* RydC due to the availability of a co-crystal structure with Hfq[8]. The residues that interact with Hfq are conserved in these homologs (see also Supplementary Table 1). Nucleotides in red were added to improve transcription by T7 polymerase. Highlights show the regions expected to bind to the proximal face (yellow), distal face (green) and rim (blue) of Hfq.

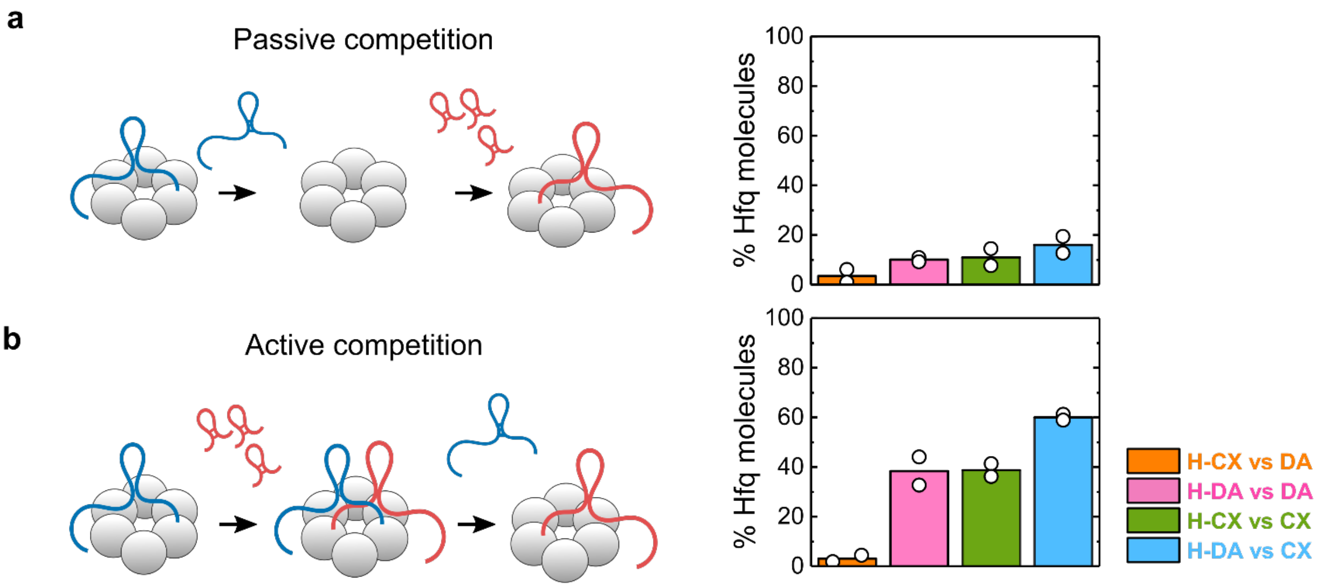

Supplementary Fig. 6. sRNAs are actively displaced from Hfq. Percentages of resident·Hfq complexes showing **a** passive and **b** active competition. The symbols indicate the results of two independent experiments. The number of molecules used to determine the percentages are reported in Supplementary Table 12. H: Hfq, DA: DsrA, CX: ChiX. Source data are provided as a Source Data file.

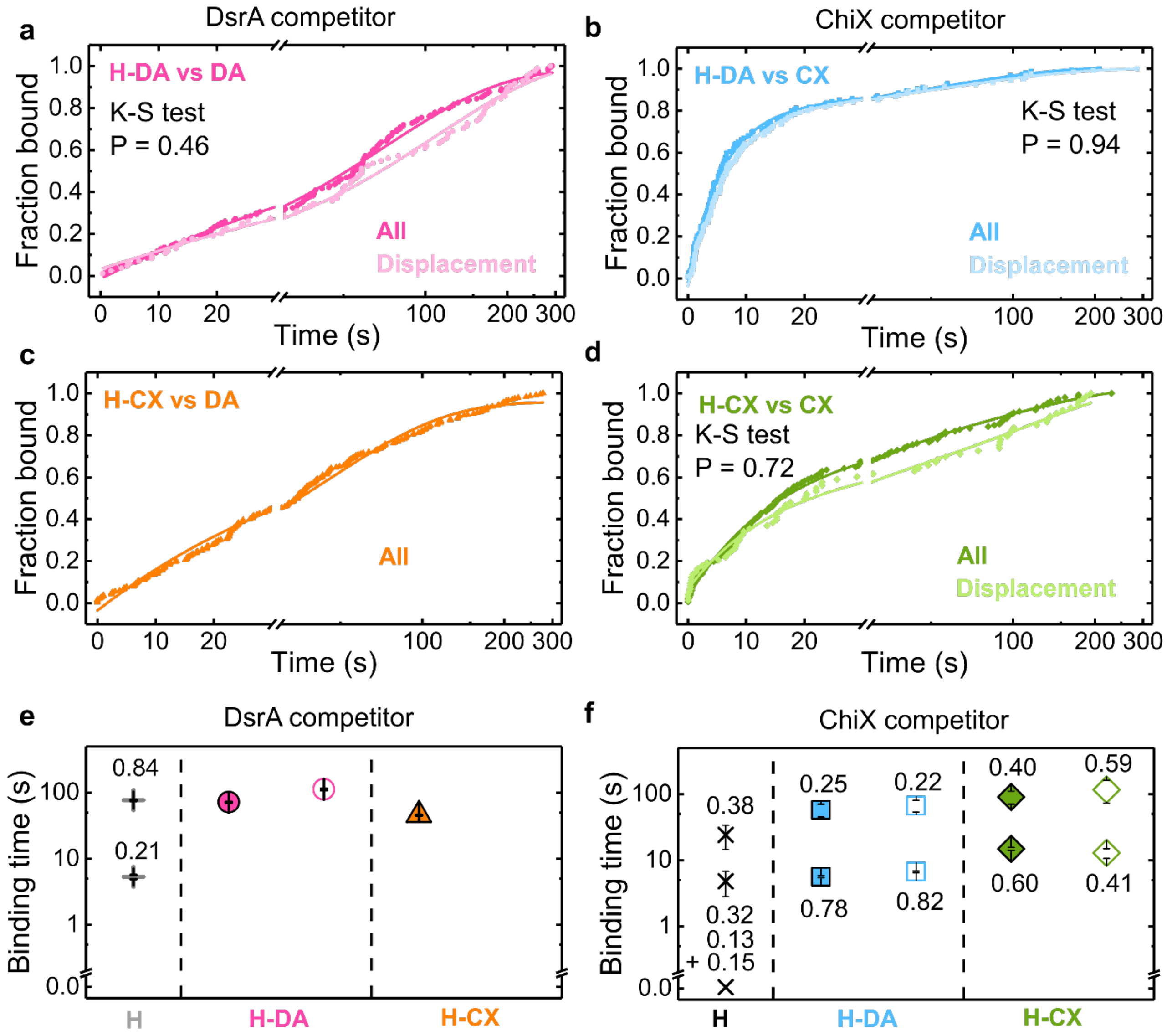

Supplementary Fig. 7. A resident on Hfq impedes competitor binding. a-d Cumulative fraction of competitor bound to resident·Hfq complexes for all types of binding (dark colors) and for binding leading to resident displacement during active competition (light colors) (See also Fig. 2a). The number of binding events for all binding categories are reported in Fig. 2. H: Hfq, DA: DsrA, CX: ChiX. The number of binding events for displacement plots are: (H-DA vs DA) = 84, (H-DA vs CX) = 119, (H-CX vs CX) = 73 from two independent experiments. Displacement for H-CX vs DA was negligible. Association functions containing one, two or three exponentials were fitted into the fraction bound vs. time to obtain binding parameters and their errors. **e-f** Parameters and errors from fits to cumulative fraction plots **a-d**: Characteristic binding times ($\tau_{bind} \propto \tau_{on}$) for **e** DsrA and **f** ChiX binding to empty

Hfq (gray and black symbols) or to sRNA·Hfq complexes (colored symbols), as indicated below the columns. Filled symbols, all binding events; open symbols, only events that led to resident displacement. Error bars were determined as the standard error from Levenberg-Marquardt non-linear curve fitting (OriginPro (2017)). The numbers next to the symbols indicate the fractions of the phases for double or triple exponential fits. Binding of ChiX to empty Hfq shows an initial 15% burst that is faster than our time resolution. (See also Supplementary Table 2). Although we don't know the origin of the complex kinetics of sRNA binding to Hfq, this may be due to encounters with different surfaces of the Hfq hexamer. It cannot be explained by different numbers of biotinylated subunits, which is one for most immobilized hexamers (Supplementary Fig. 3). Source data are provided as a Source Data file.

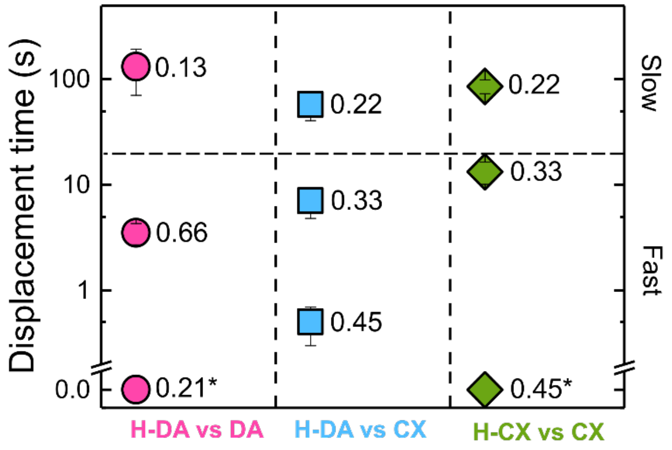

Supplementary Fig. 8. sRNA displacement is predominantly fast. Parameters and errors from fits to the probability density histogram of t_{diss} (Fig. 3c): Characteristic resident displacement times (τ_{diss}) obtained from maximum likelihood fitting to 3 exponential terms[9]; error bars are the standard deviation determined by bootstrapping of the data, as previously described[10,11]. See also Figs. 3a-c. The numbers next to the symbols report the fractions of each kinetics phase for the fits. The asterisks indicate displacement times faster than the time resolution (0.2 s) of our assay. The horizontal dashed line at 20 s separates fast from slow displacement events. H: Hfq, DA:

DsrA, CX: ChiX. (See also Supplementary Table 3). Source data are provided as a Source Data file.

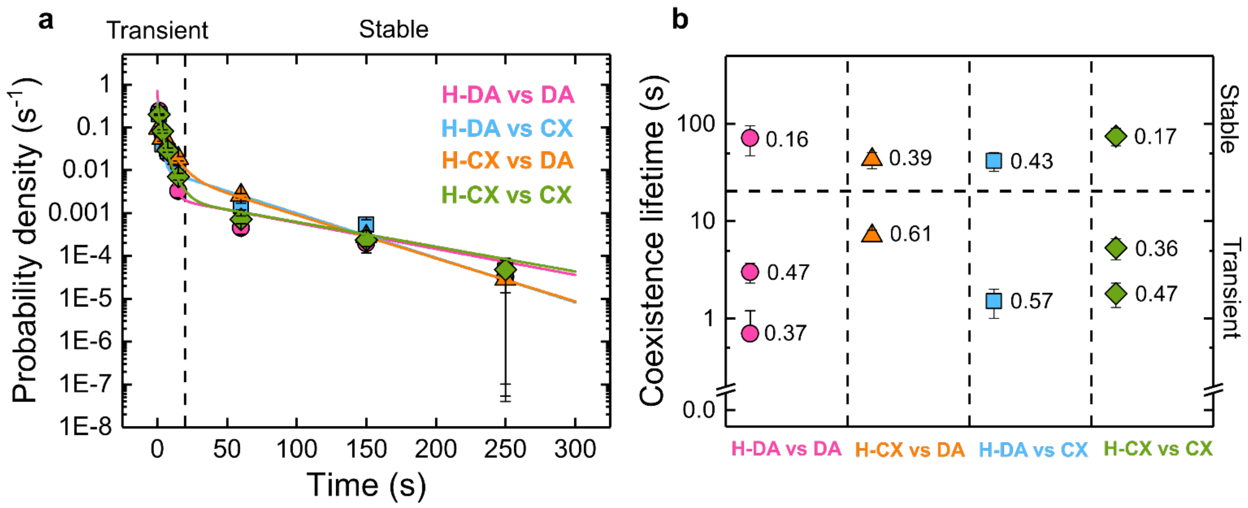

Supplementary Fig. 9. Two RNAs can coexist on Hfq transiently or stably. a Probability density histogram of coexistence times (t_{co}) of complexes containing both a resident and competitor sRNA. Solid lines represent maximum likelihood fitting to a two- or three-exponential function. Error bars represent the variance in a binomial distribution[9]. The vertical dashed line at 20 s demarcates transient from stable coexistence. The number of events/molecules for each plot are: (H-DA vs DA) = 305/142 molecules, (H-DA vs CX) = 223/154 molecules, (H-CX vs DA) = 351/93 molecules, (H-CX vs CX) = 422/151 molecules from two independent experiments. **b** Parameters and errors from fits to the probability density histogram of t_{co} **a**: Characteristic coexistence times (τ_{co}) from maximum likelihood fitting to 2 or 3 exponential terms[9]; error bars are the standard deviation determined by bootstrapping of the data, as previously described[10,11]. The numbers next to the symbols indicate the fractions of the kinetics phases. The horizontal dashed line at 20 s separates transient from stable coexistence. H: Hfq, DA: DsrA, CX: ChiX. (See also Fig. 4 and Supplementary Table 4). Source data are provided as a Source Data file.

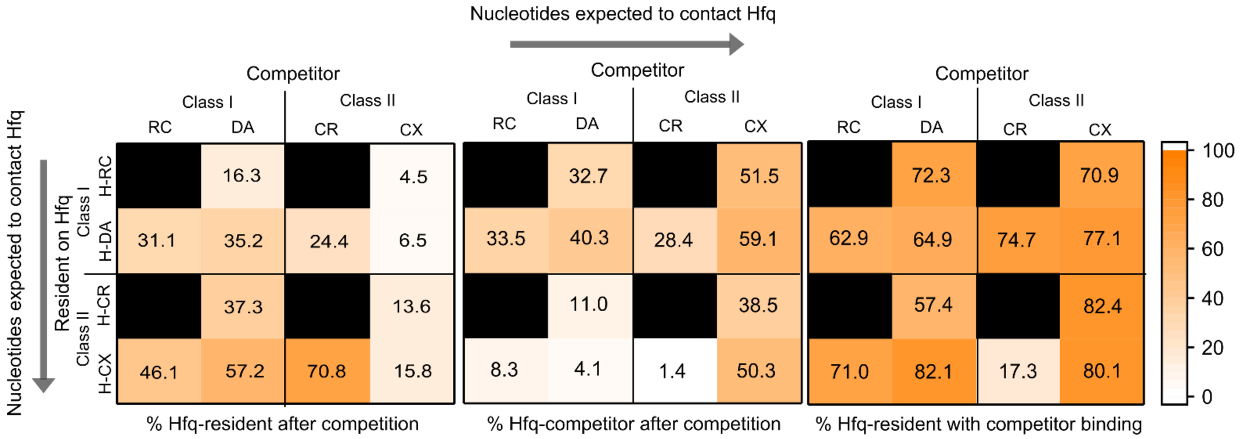

Supplementary Fig. 10. Properties of competition with class I and class II sRNAs. Heat maps depicting the percentages of (left) resident·Hfq and (middle) competitor·Hfq complexes after competition. (Right) Percentage of resident·Hfq complexes with at least one competitor binding event during real-time competition for various combinations of resident and competitor sRNAs. Percentages were calculated from the ratio of Hfq molecules showing the specified behavior over the total number of resident·Hfq complexes at the start of the experiment. Data are the mean of two independent experiments. See Supplementary Tables 9-11 for errors. The number of molecules used to determine the percentages are reported in Supplementary Table 12. H: Hfq, RC: RydC, DA: DsrA, CR: CyaR, CX: ChiX. Source data are provided as a Source Data file.

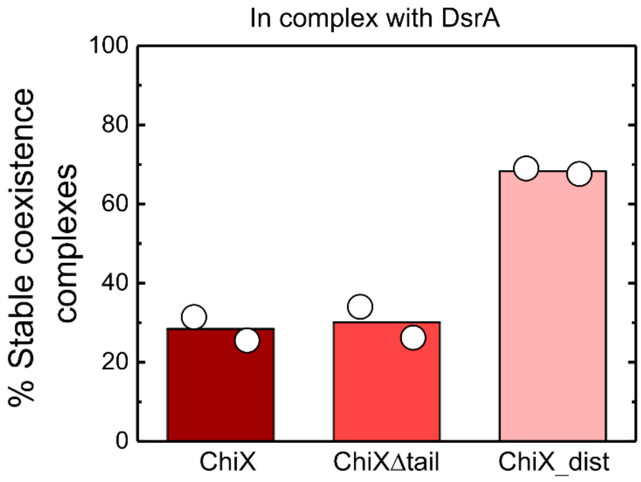

Supplementary Fig. 11. RNA binding overlap in an Hfq surface decreases stable coexistence. Percentage of stable coexistence complexes between Hfq, DsrA and variants of ChiX (Fig. 3d). The symbols indicated the results of two independent trials. ChiX, full-length; ChiXΔtail, lacking terminal uridines; ChiX_dist; 5′ fragment containing the AAN motif but not the 3′ U-rich motifs. Source data are provided as a Source Data file.

SUPPLEMENTARY TABLES

Supplementary Table 1. Sequences of the sRNAs used in this study[a]

RNA	Sequence (5'-3')
DsrA	GGGAACACAUCAGAUUUCCUGGUGUAACGAAUUUUUUAAGUGCUUCUUGCUUAAGCAAGUUUCAUCCCGACCCCCUCAGGGUCGGGAUUUUUUU
ChiX	GGACACCGUCGCUUAAAGUGACGGCAUAAUAAUAAAAAAAAUGAAAUUCCUCUUUGACGGGCCAAUAGCGAUAUUGGCCAUUUUUUU
RydC[b]	GGUUCCGAUGUAGACCCGUCCUCCUUCGCCUGCGUCACGGGUCCUGGUUAGACGCAGGCGUUUUCU
CyaR	GGCUGAAAAACAUAACCCAUAAAAUGCUAGCUGUACCAGGAACCACCUCCUUAGCCUGUGUAAUCUCCCUUACACGGGCUUAUUUUUU
ChiX_Δtail	GGACACCGUCGCUUAAAGUGACGGCAUAAUAAUAAAAAAAAUGAAAUUCCUCUUUGACGGGCCAAUAGCGAUAUUGGCCA
ChiX_dist	GGACACCGUCGCUUAAAGUGACGGCAUAAUAAUAAAAAAAAU

[a]Nucleotides in red were added to improve transcription by T7 polymerase. Unless otherwise noted, sequences are for *Escherichia coli* sRNAs.

[b]From *Salmonella enterica*. For comparison, *E. coli* RydC is shown here: CUUCCGAUGUAGACCCGUAUUCUUCGCCUGUACCACGGGUCGGUUUUAGUACAGGCGUUUUCUU[5]. Underlined nucleotides interact with Hfq and are identical in both species.

Supplementary Table 2. Characteristic competitor binding times to Hfq (τ_{bind})[a]

		Competitor				
		DsrA		ChiX[2]		
Resident		bind 2	bind 3	bind 1	bind 2	bind 3
Empty Hfq	τ_{bind} (s)	5.3 ± 0.4	76.6 ± 2.0	0.01 ± –	4.8 ± 2.0	24.1 ± 9.7
	Fraction	0.21 ± 0.01	0.84 ± 0.01	0.13 ± 0.02	0.32 ± 0.13	0.38 ± 0.12
DsrA	τ_{bind} (s)		71.4 ± 1.1		5.6 ± 0.2	57.5 ± 12.6
	Fraction				0.78 ± 0.02	0.25 ± 0.01
DsrA w/diss[1]	τ_{bind_diss} (s)		111.9 ± 3.1		6.7 ± 0.2	66.5 ± 13.3
	Fraction				0.82 ± 0.02	0.22 ± 0.01
ChiX	τ_{bind} (s)		45.6 ± 0.7		14.8 ± 0.8	90.5 ± 20.6
	Fraction				0.60 ± 0.04	0.40 ± 0.02
ChiX w/diss[1]	τ_{bind_diss} (s)				12.8 ± 2.2	117.1 ± 44.3
	Fraction				0.41 ± 0.07	0.59 ± 0.04

[a]The data were fit with rate equations containing 1, 2 or 3 exponential terms (as indicated in the top row) to obtain the parameters and their errors (see Methods). "–", the error could not be estimated from the fit. The fraction represents the proportion of the population or amplitude of each phase.

[1]Characteristic competitor binding times for events in which sRNA residents were displaced from Hfq (τ_{bind_diss}). Displacement when ChiX is a resident and DsrA a competitor was negligible and not included in the analysis.

[2]Binding of ChiX to empty Hfq also shows an initial burst of 0.15, indicating a phase faster than our time resolution.

Supplementary Table 3. Characteristic resident displacement times from Hfq (τ_{diss})[a]

			Competitor					
			DsrA			ChiX[2]		
			diss 1	diss 2	diss 3	diss 1	diss 2	diss 3
Resident	DsrA	τ_{diss} (s)	≤ 0.2 ± -	3.5 ± 0.8	132.5 ± 61.3	0.5 ± 0.2	7.1 ± 2.3	57.9 ± 17.2
		Fraction	0.21 ± 0.12	0.66 ± 0.07	0.13 ± 0.14	0.45 ± 0.05	0.33 ± 0.04	0.22 ± 0.06
	ChiX[1]	τ_{diss} (s)				≤ 0.2 ± -	13.3 ± 3.1	85.9 ± 13.0
		Fraction				0.45 ± 0.04	0.33 ± 0.02	0.22 ± 0.04

[a]The data were fit using maximum likelihood with 1, 2 or 3 exponential terms (as indicated in the row "Kinetics phase") to obtain the parameters. Errors were obtained by bootstrapping (see Methods). "–", the error could not be estimated from the fit. Values shown as ≤ 0.2 were reported as 0 by maximum likelihood analysis. These lifetimes are likely faster than the time resolution of the experiment (0.2 s).

[1]Displacement when ChiX is a resident and DsrA a competitor was negligible and not included in the analysis.

Supplementary Table 4. Characteristic coexistence times of resident and competitor sRNAs on Hfq (τ_{co})[a]

			Competitor					
			DsrA			ChiX[2]		
			co 1	co 2	co 3	co 1	co 2	co 3
Resident	DsrA	τ_{co} (s)	0.7 ± 0.5	3.0 ± 0.7	71.4 ± 24.2	1.5 ± 0.5		41.8 ± 9.4
		Fraction	0.37 ± 0.10	0.47 ± 0.09	0.16 ± 0.10	0.57 ± 0.05		0.43 ± 0.05
	ChiX	τ_{co} (s)		7.1 ± 1.0	43.0 ± 8.1	1.8 ± 0.5	5.3 ± 1.3	75.2 ± 15.3
		Fraction		0.61 ± 0.04	0.39 ± 0.04	0.47 ± 0.09	0.36 ± 0.08	0.17 ± 0.12

[a]The data were fit using maximum likelihood with 1, 2 or 3 exponential terms (as indicated in the row "Kinetics phase") to obtain the parameters. Errors were obtained by bootstrapping (see Methods).

Supplementary Table 5. Ranking of sRNAs according to their possible interactions with Hfq

	Number of nucleotides expected to interact with Hfq			
sRNA	With proximal (U tail)	With distal (ARN motifs)	With rim	Total
RydC	5	0	4	9
DsrA	6	0	10	16
CyaR	6	12	3	21
ChiX	7	12	6	25

[a]A: Adenine, R: Purine, N: Any nucleotide.

Supplementary Table 6. Percentage of resident·Hfq molecules showing passive competition[a]

Resident		Competitor: RydC	Competitor: DsrA	Competitor: CyaR	Competitor: ChiX
	RydC		10.7 | 5.3<br>8.0 ± 2.7		18.0 | 2.0<br>10.0 ± 8.0
	DsrA	23.7 | 9.9<br>16.8 ± 6.9	10.9 | 9.2<br>10.1 ± 0.9	8.1 | 4.9<br>6.5 ± 1.6	19.4 | 12.7<br>16.1 ± 3.4
	CyaR		6.4 | 9.0<br>7.7 ± 1.3		6.1 | 3.8<br>5.0 ± 1.2
	ChiX	1.9 | 4.9<br>3.4 ± 1.5	6.1 | 0.9<br>3.5 ± 2.6	3.0 | 2.5<br>2.8 ± 0.3	7.6 | 14.4<br>11.0 ± 3.4

[a]Top values correspond to two independent experiments; bottom values indicate their average; errors are the s.e.m.

Supplementary Table 7. Percentage of resident·Hfq molecules showing stable coexistence[a]

Resident	Competitor:	RydC	DsrA	CyaR	ChiX
	RydC		27.2 | 17.0<br>22.1 ± 5.1		23.0 | 45.1<br>34.1 ± 11.1
	DsrA	29.9 | 15.8<br>22.9 ± 7.1	11.8 | 13.8<br>12.8 ± 1.0	45.5 | 58.8<br>52.2 ± 6.7	25.5 | 31.4<br>28.5 ± 3.0
	CyaR		40.9 | 29.0<br>35.0 ± 6.0		44.9 | 72.4<br>58.7 ± 13.8
	ChiX	48.1 | 52.9<br>50.5 ± 2.4	48.3 | 60.6<br>54.5 ± 6.2	1.0 | 4.2<br>2.6 ± 1.6	46.7 | 26.8<br>36.8 ± 10.0

[a]Top values correspond to two independent experiments; bottom values indicate their average; errors are the s.e.m.

Supplementary Table 8. Percentage of resident·Hfq molecules showing active competition[a]

Resident	Competitor →	RydC	DsrA	CyaR	ChiX
	RydC		42.7 | 34.0<br>38.4 ± 4.4		60.0 | 53.9<br>57.0 ± 3.1
	DsrA	32.1 | 23.8<br>28.0 ± 4.2	32.7 | 44.0<br>38.4 ± 5.7	37.4 | 40.2<br>38.8 ± 1.4	61.2 | 58.8<br>60.0 ± 1.2
	CyaR		10.0 | 8.0<br>9.0 ± 1.0		39.8 | 56.2<br>48.0 ± 8.2
	ChiX	6.7 | 7.8<br>7.3 ± 0.6	4.4 | 1.8<br>3.1 ± 1.3	1.0 | 0.0<br>0.5 ± 0.5	41.3 | 36.1<br>38.7 ± 2.6

[a]Top values correspond to two independent experiments; bottom values indicate their average; errors are the s.e.m.

Supplementary Table 9. Percentage of resident·Hfq molecules after competition[a]

		Competitor			
		RydC	DsrA	CyaR	ChiX
Resident	RydC		16.5 | 16.0<br>16.3 ± 0.3		5.0 | 3.9<br>4.5 ± 0.6
	DsrA	20.6 | 41.5<br>31.1 ± 10.5	38.2 | 32.1<br>35.2 ± 3.1	26.3 | 22.5<br>24.4 ± 1.9	6.1 | 6.9<br>6.5 ± 0.4
	CyaR		35.5 | 39.0<br>37.3 ± 1.8		20.4 | 6.7<br>13.6 ± 6.9
	ChiX	47.1 | 45.1<br>46.1 ± 1.0	52.8 | 61.5<br>57.2 ± 4.4	71.0 | 70.6<br>70.8 ± 0.2	13.0 | 18.6<br>15.8 ± 2.8

[a]Top values correspond to two independent experiments; bottom values indicate their average; errors are the s.e.m.

Supplementary Table 10. Percentage of competitor·Hfq molecules after competition[a]

Resident \ Competitor	RydC	DsrA	CyaR	ChiX
RydC		40.8 | 24.5<br>32.7 ± 8.2		51.0 | 52.0<br>51.5 ± 0.5
DsrA	41.2 | 25.7<br>33.5 ± 7.8	35.5 | 45.0<br>40.3 ± 4.8	25.3 | 31.4<br>28.4 ± 3.1	65.3 | 52.9<br>59.1 ± 6.2
CyaR		10.9 | 11.0<br>11.0 ± 0.1		26.5 | 50.5<br>38.5 ± 12.0
ChiX	6.7 | 9.8<br>8.3 ± 1.6	7.2 | 0.9<br>4.1 ± 3.2	2.0 | 0.8<br>1.4 ± 0.6	51.1 | 49.5<br>50.3 ± 0.8

[a]Top values correspond to two independent experiments; bottom values indicate their average; errors are the s.e.m.

Supplementary Table 11. Percentage of resident·Hfq showing competitor binding[a]

Resident		Competitor: RydC	Competitor: DsrA	Competitor: CyaR	Competitor: ChiX
	RydC		79.6 | 64.9<br>72.3 ± 7.4		76.0 | 65.7<br>70.9 ± 5.2
	DsrA	73.2 | 52.5<br>62.9 ± 10.4	60.9 | 68.8<br>64.9 ± 4.0	75.8 | 73.5<br>74.7 ± 1.2	77.6 | 76.5<br>77.1 ± 0.6
	CyaR		61.8 | 53.0<br>57.4 ± 4.4		72.4 | 92.4<br>82.4 ± 10.0
	ChiX	62.5 | 79.4<br>71.0 ± 8.5	78.9 | 85.3<br>82.1 ± 3.2	17.0 | 17.6<br>17.3 ± 0.3	84.8 | 75.3<br>80.1 ± 4.8

[a]Top values correspond to two independent experiments; bottom values indicate their average; errors are the s.e.m.

Supplementary Table 12. Number of molecules used to determine the averages of active, soft and passive competition, resident·Hfq and competitor·Hfq complexes after competition and resident·Hfq complexes with at least one competitor binding (See Supplementary Tables 6-11)[a]

Resident		Competitor			
		RydC	DsrA	CyaR	ChiX
	RydC		103 | 94		100 | 102
	DsrA	97 | 101	110 | 109	99 | 102	98 | 102
	CyaR		110 | 100		98 | 105
	ChiX	104 | 102	180 | 109	100 | 119	92 | 97

[a]Values correspond to two independent experiments.

Supplementary Table 13. Sequences of primers used in this study

Primer	Sequence (5’-3’)
Set 1: Hfq102_CAvi-F	GGCCTGAACGACATCTTCGAGGCTCAGAAAATCGAATGGC ACGA ATAAGCCGAATTCGAGCTC
Set 1: Hfq102_CAvi-R	TTCGGTTTCTTCGCTGTC
Set 2: Hfq_rha1-F	AAGCTCTTCTATGGCTAAGGGGC
Set 2: Hfq-rhaC-R	ACGGCTCTTCTACCTTATTCGTGC
Set 3: Hfq_rha1-F	AAGCTCTTCTATGGCTAAGGGGC
Set 3: Hfq_rhaA-R	ACGGCTCTTCTACCTTATTCGGTTTC

SUPPLEMENTARY REFERENCES

1. Santiago-Frangos, A., Kavita, K., Schu, D. J., Gottesman, S. & Woodson, S. A. C-terminal domain of the RNA chaperone Hfq drives sRNA competition and release of target RNA. *Proc. Natl. Acad. Sci.* **113**, E6089–E6096 (2016).
2. Brescia, C. C. & Mikulecky, P. J. Identification of the Hfq-binding site on DsrA RNA: Hfq binds without altering DsrA secondary structure. *RNA* **9**, 33–43 (2003).
3. Fratczak, A., Kierzek, R. & Kierzek, E. Isoenergetic microarrays to study the structure and interactions of DsrA and OxyS RNAs in two- and three-component complexes. *Biochemistry* **50**, 7647–7665 (2011).
4. Rasmussen, A. A. *et al.* A conserved small RNA promotes silencing of the outer membrane protein YbfM. *Mol. Microbiol.* **72**, 566–577 (2009).
5. Antal, M., Bordeau, V., Douchin, V. & Felden, B. A small bacterial RNA regulates a putative ABC transporter. *J. Biol. Chem.* **280**, 7901–7908 (2005).
6. Fröhlich, K. S., Papenfort, K., Fekete, A. & Vogel, J. A small RNA activates CFA synthase by isoform-specific mRNA stabilization. *EMBO J.* **32**, 2963–2979 (2013).
7. Zuker, M. Mfold web server for nucleic acid folding and hybridization prediction. *Nucleic Acids Res.* **31**, 3406–3415 (2003).
8. Dimastrogiovanni, D. *et al.* Recognition of the small regulatory RNA RydC by the bacterial Hfq protein. *Elife* **3**, 1–19 (2014).
9. Friedman, L. J. & Gelles, J. Multi-wavelength single-molecule fluorescence analysis of transcription mechanisms. *Methods* **86**, 27–36 (2015).
10. Rodgers, M. L. & Woodson, S. A. Transcription increases the cooperativity of ribonucleoprotein assembly. *Cell* **179**, 1370–1381 (2019).
11. Małecka, E. M. & Woodson, S. A. Stepwise sRNA targeting of structured bacterial mRNAs leads to abortive annealing. *Mol. Cell* **81**, 1988-1999.e4 (2021).
