# Supplementary material for: Diversity of bacterial small RNAs drives competitive strategies for a mutual chaperone
Source: Nat Commun. 2022 May 4;13:2449. doi: 10.1038/s41467-022-30211-z (PMC9068810; doi:10.1038/s41467-022-30211-z)
Supplement: Supplementary file 3 — Reporting Summary [file 41467_2022_30211_MOESM3_ESM.pdf]

nature portfolio

Corresponding author(s): Sarah A. Woodson

Last updated by author(s): Mar 8, 2022

Reporting Summary

Nature Portfolio wishes to improve the reproducibility of the work that we publish. This form provides structure for consistency and transparency in reporting. For further information on Nature Portfolio policies, see our Editorial Policies and the Editorial Policy Checklist.

Statistics

For all statistical analyses, confirm that the following items are present in the figure legend, table legend, main text, or Methods section.

n/a	Confirmed	
☐	☒	The exact sample size (*n*) for each experimental group/condition, given as a discrete number and unit of measurement
☐	☒	A statement on whether measurements were taken from distinct samples or whether the same sample was measured repeatedly
☐	☒	The statistical test(s) used AND whether they are one- or two-sided<br>*Only common tests should be described solely by name; describe more complex techniques in the Methods section.*
☒	☐	A description of all covariates tested
☒	☐	A description of any assumptions or corrections, such as tests of normality and adjustment for multiple comparisons
☐	☒	A full description of the statistical parameters including central tendency (e.g. means) or other basic estimates (e.g. regression coefficient) AND variation (e.g. standard deviation) or associated estimates of uncertainty (e.g. confidence intervals)
☒	☐	For null hypothesis testing, the test statistic (e.g. *F*, *t*, *r*) with confidence intervals, effect sizes, degrees of freedom and *P* value noted<br>*Give P values as exact values whenever suitable.*
☒	☐	For Bayesian analysis, information on the choice of priors and Markov chain Monte Carlo settings
☒	☐	For hierarchical and complex designs, identification of the appropriate level for tests and full reporting of outcomes
☒	☐	Estimates of effect sizes (e.g. Cohen's *d*, Pearson's *r*), indicating how they were calculated

Our web collection on statistics for biologists contains articles on many of the points above.

Software and code

Policy information about availability of computer code

Data collection	Single-molecule fluorescence data were acquired using the smCamera software available at https://github.com/Ha-SingleMoleculeLab/Data-Aquisition.
Data analysis	Processing of single-molecule data was done with the Imscroll software available at https://github.com/gelles-brandeis/CoSMoS_Analysis.<br>Cumulative fraction bound plots, binding and competition curves were fitted with OriginPro 2017 (b9.4.2.380).<br>Gel images were quantified using ImageJ (1.53c).<br>Because these codes were not written by us and are already publicly available, we supplied a statement that no new code is associated with this publication.

For manuscripts utilizing custom algorithms or software that are central to the research but not yet described in published literature, software must be made available to editors and reviewers. We strongly encourage code deposition in a community repository (e.g. GitHub). See the Nature Portfolio guidelines for submitting code & software for further information.

Data

Policy information about availability of data

All manuscripts must include a data availability statement. This statement should provide the following information, where applicable:

- Accession codes, unique identifiers, or web links for publicly available datasets
- A description of any restrictions on data availability
- For clinical datasets or third party data, please ensure that the statement adheres to our policy

Statement in the paper: The data that support the findings of this study are available from the corresponding author upon reasonable request. Source data are provided with this paper.

There is presently no public curated repository for single molecule data, and the ~80GB raw data would not be useful even for experts without an intimate knowledge of how the data were collected. At present, single molecule data are not typically deposited in open databases. For this reason, we prefer to make the data available upon request (without restrictions).

Field-specific reporting

Please select the one below that is the best fit for your research. If you are not sure, read the appropriate sections before making your selection.

☒ Life sciences ☐ Behavioural & social sciences ☐ Ecological, evolutionary & environmental sciences

For a reference copy of the document with all sections, see nature.com/documents/nr-reporting-summary-flat.pdf

Life sciences study design

All studies must disclose on these points even when the disclosure is negative.

Sample size	All the experiments were independently repeated with different samples at least two times. For single-molecule experiments, at least 100 molecules from each independent experiment were selected for analysis. This number of molecules used is consistent with similar experiments in the field and reasonable to obtain enough events to create curves and histograms amenable for fitting.
Data exclusions	No data were excluded.
Replication	All the experiments were independently repeated at least two times. All attempts at replication were successful.
Randomization	We did not allocate samples into experimental groups, and therefore randomization of such allocation was not required.
Blinding	During single molecule or other experiments the investigators were unaware of the outcome of the experiments. In addition to it, we did not use any group allocation and hence, blinding was not relevant to this study.

Reporting for specific materials, systems and methods

We require information from authors about some types of materials, experimental systems and methods used in many studies. Here, indicate whether each material, system or method listed is relevant to your study. If you are not sure if a list item applies to your research, read the appropriate section before selecting a response.

Materials & experimental systems

n/a	Involved in the study	
☐	☒	Antibodies
☒	☐	Eukaryotic cell lines
☒	☐	Palaeontology and archaeology
☒	☐	Animals and other organisms
☒	☐	Human research participants
☒	☐	Clinical data
☒	☐	Dual use research of concern

Methods

n/a	Involved in the study	
☒	☐	ChIP-seq
☒	☐	Flow cytometry
☒	☐	MRI-based neuroimaging

Antibodies

Antibodies used	Avi Tag Antibody, mAb, Mouse. Supplier: GenScript. Cat. No: A01738-40. Clone: 1D11D10. Lot No: H2103013. 1:1000 dilution.<br>Alexa Fluor 594 goat anti-mouse antibody. Supplier: Invitrogen. Cat. No: R37121. Polyclonal. Lot No: 1856554. 1:5000 dilution.
Validation	The antibodies were tested with purified Hfq and Hfq-CAvi as shown in the Western blot images in the Supplementary Information.
